# A Comparative Study of Urinary Tests and Cultures for the Effectiveness of Fosfomycin in Catheter-Related Urinary Tract Infections

**DOI:** 10.3390/jcm11237229

**Published:** 2022-12-05

**Authors:** Jung Ki Jo, Dong Seob Kim, Younghun Sim, Soorack Ryu, Kyu Shik Kim

**Affiliations:** 1 Department of Urology, College of Medicine, Hanyang University, Seoul 04763, Republic of Korea; 2 Biostatistical Consulting and Research Laboratory, Medical Research Collaborating Center, Hanyang University, Seoul 04763, Republic of Korea; 3 Department of Urology, Hanyang University Guri Hospital, Gyeonggi-do 11923, Republic of Korea

**Keywords:** catheter, fosfomycin, urinary tract infection, urine culture

## Abstract

As the elderly population increases due to an aging society, the number of patients with catheters is increasing, and treatment for urinary infections is needed. The current study analyzed the effectiveness of fosfomycin, the primary antibiotic used to treat urinary tract infections (UTIs), in these patients. Patients who received fosfomycin as the primary antibiotic for a UTI were selected, and the results of urine tests and cultures before and after fosfomycin administration were compared and analyzed. The degree of UTI in patients with a catheter was found to be more severe (*p* = 0.020), and the infecting strains were found to be different depending on whether a catheter was present (*p* = 0.014). There was a difference in the treatment success rate depending on whether or not a catheter was present (53.6% vs. 70.4%), but it was found that the treatment rate was more than 50% regardless of whether a catheter was present. The bacterial type, as well as the treatment rate based on the bacterium, differed depending on the presence of a catheter. Fosfomycin has a success rate of more than 50%, even in patients with catheters; therefore, it can be considered the primary antibiotic for treating UTIs.

## 1. Introduction

Urinary tract infections (UTIs) associated with catheter use are common. Urinary catheter use is linked to the development of 70–80% of hospital-acquired UTIs [1,2,3], and the daily risk of bacteriuria related to catheterization ranges from 3–10% [4]. Catheter-associated UTIs in surgical patients are estimated to cost USD 558 to USD 676 per patient [3,5]. Surgical patients receiving prophylactic antibiotics have a lower incidence of bacteriuria, pyuria, febrile morbidity, and Gram-negative isolates according to a Cochrane review of randomized controlled trials [6]. However, if antibiotic treatment is abused, there is a risk that bacteria in the urinary system can become resistant to antibiotics, which can lead to UTIs. Currently, no agreement has been reached on the use of prophylactic antibiotics in patients with short-term in-dwelling urinary catheters, and there has been no investigation into the cost-effectiveness of these therapies.

When comparing the costs and benefits of various approaches, cost-effectiveness analysis is conducted using decision tree models to perform comparisons. The goal of a decision tree model is to consider all the probabilities, costs, and impacts of various strategies to establish the circumstances under which one strategy is more cost-effective than another.

This study aimed to examine the effectiveness of fosfomycin in treating catheter-associated UTIs. The results of the urine culture tests were used to assess the percentage of resistant bacteria.

## 2. Materials and Methods

### 2.1. Study Cohort

Over the course of the study, 1000 patients who were approved by the Institutional Review Board and administered fosfomycin, the first-line treatment for UTIs based on large-scale epidemiological research, were retrospectively evaluated. Patients whose medical data were not accessible or those who were taking other medications were excluded. Finally, 433 participants were included in the study and were categorized according to whether or not a catheter was present.

### 2.2. Primary Outcome

The results of urine tests and cultures performed both before and after fosfomycin administration were examined to determine the effectiveness of the treatment for each patient, considering whether or not a catheter was used throughout the treatment. Like other urinary tract infections, empirical antibiotics are administered, and in principle, urine tests and cultures are performed before antibiotics are administered.

#### 2.2.1. Routine Urine Analysis (RUA)

RUA with a white blood cell count of 5–9 per mm^3^ or higher was associated with pyuria, whereas analysis at postvention showed a white blood cell count of 1–4 per mm^3^. In the pretreatment urine test, bacteriuria was labeled as few, moderate, or many bacteria, and therapy was considered successful when the post-treatment test showed no bacteria. 

#### 2.2.2. Urine Culture

RRE C bacteria names were given to the microorganisms. The purpose of this experiment was to determine the effectiveness of the therapy in relation to the presence or absence of a catheter using strain. Each RRE C bacteria name, for instance, Escherichia coli and Candida glabrata, was classified as treated when the postC bacteria name was less than 1000 or there was no growth. This was the case, regardless of whether there was growth.

#### 2.2.3. Sensitivity Test

Investigations were conducted to demonstrate the differences in antibiotic sensitivity in relation to the presence or absence of a catheter. As part of this study, we investigated whether fosfomycin could be used as the primary antibiotic for patients undergoing catheterization.

### 2.3. Statistics

In this investigation, each test was conducted on both sets of data, and statistical significance was set at *p*-value < 0.05. Statistical analysis was performed using SAS version 9.4 (SAS Institute, Cary, NC, USA), and the results were interpreted using the SAS system. The R program was used to perform statistical analysis using version 4.0.4 (R Project for Statistical Computing).

## 3. Results

### 3.1. Characteristics of the Cohort

During this research project, 433 patients were administered fosfomycin for the treatment of UTIs. Of these 433 patients, 86 used a catheter, whereas the remaining 347 patients did not. There were no differences in age, sex, or prevalence of diabetes; however, the hospitalization periods were different among the groups (Table 1).

### 3.2. Urine Test

Table 2 presents the findings of the urine test before treatment, according to the presence of a catheter. The results demonstrated that there was a difference between the two groups regarding turbidity, microscopic hematuria status (red blood cells status), and the presence of bacteria. Turbidity was found to be significantly higher in the group that used a catheter (*p* < 0.001). Additionally, microscopic hematuria and the presence of bacteria were found to be significantly more common in the group before treatment (*p* < 0.001 and *p* = 0.02, respectively).

### 3.3. Urine Culture

There was a distinction between the two groups in terms of the variety of bacteria that were cultivated (*p* = 0.014; Table 3). In the group that did not use catheters, *E. coli* was the most commonly isolated bacteria in the urine cultures. However, in the group that used catheters, several other species of bacteria besides *E. coli* were cultivated. These bacteria included the Enterobacter and Enterococcus species.

### 3.4. Proportion of Successful Treatments

The percentage of patients who responded well to fosfomycin treatment in both groups is shown in Table 4. Urine tests showed that the catheter-free group was more likely to improve after treatment in terms of turbidity, pH, pyuria, microscopic hematuria, and bacterial status. Table 5 provides a summary of the treatment success rate according to the distribution of diabetes, hypertension, and other conditions that need catheterization.

### 3.5. Results of Antibiotic Sensitivity

**Figure 1:**
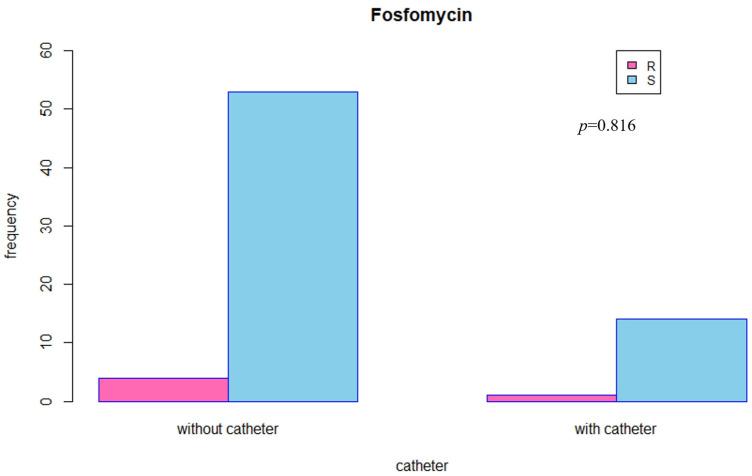
Fosfomycin antibiotics sensitivity according to catheter group.

The findings of the antibiotic sensitivity tests are shown in Table 6, organized according to catheter status. The findings of this investigation indicated that there was no discernible difference in the levels of antibiotic resistance between the two groups. Additionally, there was no discernible difference in the levels of fosfomycin resistance between the two groups (*p* = 0.816) (Figure 1). 

## 4. Discussion

Within the scope of this study, we investigated the prevalence of bacterial infections and the effectiveness of fosfomycin in the treatment of UTIs caused by urethral catheters. By performing a urine test and analyzing the results, we were able to demonstrate the disparity between the urine test results and the distribution of infectious bacteria between urinary tract infections caused by urinary catheters and other types of urinary tract infections. An increase in the prevalence of antibiotic-resistant UTIs is one of the primary causes for concern when it comes to infections associated with catheters. During the sensitivity analysis of the urine culture test, we focused on the efficiency of fosfomycin in comparison to other readily accessible antibiotics. According to the results of our study, fosfomycin is effective even in patients with catheters, even if judged based on urine culture tests.

Previous research has demonstrated that antibiotic prophylaxis prior to catheter insertion for a short period after surgery lowers the risk of catheter-related UTIs. Efforts have also been made to describe the financial repercussions of catheter-related urinary tract infections. The current body of research has reported several different antibiotic resistance rates, and, with this objective in mind, our sensitivity analysis examined several different antibiotic resistance rates. However, these findings should not be used to make decisions about individual patients unless they are interpreted in the context of established therapeutic standards.

In addition, the approach should consider the patient’s medical history, comorbidities, and personal preferences. In the current study, we performed an assessment to determine whether fosfomycin is an effective antibiotic for the treatment and prevention of urinary tract infections caused by urethral catheters.

*E. coli* was identified as the most prevalent pathogen in patients with UTIs using urethral catheters. Individuals who have undergone short-term catheterization typically have Pseudomonas, Klebsiella, Proteus, Enterococcus, and Candida species in their systems. Patients who use catheters for extended periods have an increased risk of developing polymicrobial bacteriuria, and bacteriuria caused by Proteus mirabilis is commonly associated with catheter obstruction [2,7].

Fosfomycin is an attractive option for treating UTIs because of its quick absorption after oral administration, its concentration for elimination in urine, its effectiveness against biofilms [8,9], and its ability to combat a wide variety of multidrug-resistant pathogens, including extended-spectrum beta-lactamase and AmpC-producing bacteria of the family Enterobacteriaceae [10]. Oral fosfomycin is well tolerated and, for the most part, is devoid of major adverse side effects [11,12]. Only 5% of patients reported side effects, with the most common being diarrhea [13].

Fosfomycin dosing approaches can vary. The National Institute for Health and Care Excellence guidelines recommend a single dose of 3 g for women and two doses of 3 g (at an interval of 3 days) for men [14], but the United Kingdom (UK) product license is only for a single dose, and the European guidelines formulated by the European Association of Urology do not recommend that men use fosfomycin [15]. Although the UK recommendation is limited to uncomplicated UTIs [14,16,17,18] and existing guidance focuses on out-patient treatment, the administration of fosfomycin has also shown reasonable success in patients with risk factors for persistent or recurrent UTIs [11,13,19].

This study had certain limitations. First, it was a retrospective data analysis. Second, it was not structured as large-scale research, and finally, there was no further investigation of the economic benefits. However, a recent large-scale epidemiological study has recommended fosfomycin for the treatment of UTIs. This study is important because it is the first to determine the effectiveness of fosfomycin in treating UTIs caused by catheters. In the future, this should be tested using large-scale prospective comparative analytical research.

## 5. Conclusions

In this study, urine cultures were performed and the results showed that the presence or absence of a catheter resulted in distinct patterns of bacterial infection, which could be validated by examining the bacteria that were present. Even in patients using catheters, fosfomycin may be regarded as the primary antibiotic because the treatment success rate of fosfomycin for UTIs was higher than 50% in all cases.

## Figures and Tables

**Table 1:** Characteristics of study cohort.

	Catheter Group	Non-Catheter Group	*p*-Value
Patient number	86	347	
Age	68.03 ± 17.85	65.25 ± 17.46	0.188
Gender			
Male	22 (25.6%)	64 (18.4%)	0.138
female	64 (74.4%)	283 (81.6%)	
DM ^1^			
DM (−)	62 (72.1%)	258 (74.4%)	0.669
DM (+)	24 (27.9%)	89 (25.7%)	
HTN ^2^			
HTN (−)	34 (39.5%)	182 (52.5%)	0.032
HTN (+)	52 (60.5%)	165 (47.6%)	
Days of administration	2.52 ± 2.22	3.26 ± 3.00	0.012

^1^ DM: Diabetes Melitus, ^2^ HTN: Hypertension.

**Table 2:** The results of the urine tests before treatment.

	Catheter Group (N = 86)	Non-Catheter Group (N = 347)	*p*-Value
Pre_Turbidity			
Clear	35 (40.7%)	212 (61.1%)	<0.001
Hazy	4 (4.7%)	31 (8.9%)	
Light turbid	27 (31.4%)	58 (16.7%)	
Turbid	20 (23.3%)	46 (13.3%)	
Pre_pH			
Abnormal	0 (0.0%)	2 (0.6%)	0.4804
Normal	86 (100.0%)	345 (99.4%)	
Pre_WBC ^1^			
0–1	1 (1.2%)	11 (3.2%)	0.396
0–2	5 (5.8%)	38 (11.0%)	
1–4	1 (1.2%)	14 (4.0%)	
3–4	5 (5.8%)	28 (8.1%)	
5–9	8 (9.3%)	33 (9.5%)	
10–19	15 (17.4%)	45 (13.0%)	
20–29	13 (15.1%)	37 (10.7%)	
Many	38 (44.2%)	141 (40.6%)	
Pre_RBC ^2^			
0–1	1 (1.2%)	18 (5.2%)	<0.001
0–2	20 (23.3%)	132 (38.0%)	
1–4	6 (7.0%)	62 (17.9%)	
3–4	10 (11.6%)	26 (7.5%)	
5–9	11 (12.8%)	34 (9.8%)	
10–19	9 (10.5%)	25 (7.2%)	
20–29	5 (5.8%)	9 (2.6%)	
Many	24 (27.9%)	41 (11.8%)	
Pre_Bacteria			
<1000 UFC/mL	18 (20.9%)	121 (34.9%)	0.020
A few	12 (14.0%)	64 (18.4%)	
Moderate	28 (32.6%)	76 (21.9%)	
Many	28 (32.6%)	86 (24.8%)	

^1^ WBC: White blood cell, ^2^ RBC: Red blood cell.

**Table 3:** The results of the urine cultures before treatment.

RRE C ^1^ Bacteria Name	Catheter Group (N = 16)	Non-Catheter Group (N = 65)	*p*-Value
*Citrobacter*	0 (0.0%)	1 (100.0%)	0.022
*Enterobacter*	2 (66.7%)	1 (33.3%)	
*Enterococcus*	2 (33.3%)	4 (66.7%)	
*Escherichia coli*	3 (10.7%)	25 (89.3%)	
*Escherichia faecalis*	1 (100.0%)	0 (0.0%)	
*Etreptococcus*	0 (0.0%)	1 (100.0%)	
*Klebsiella*	1 (16.7%)	5 (83.3%)	
*Less than 1000*	3 (12.0%)	22 (88.0%)	
*Proteus mirabilis*	0 (0.0%)	1 (100.0%)	
*Pseudomonas*	1 (100.0%)	0 (0.0%)	
*Staphylococcus*	0 (0.0%)	1 (100.0%)	
*Streptococcus*	0 (0.0%)	2 (100.0%)	
*Yeast*	1 (100.0%)	0 (0.0%)	
*Acinetobacter baumannii*	0 (0.0%)	2 (100.0%)	
*Aerococcus viridans*	1 (100.0%)	0 (0.0%)	

^1^ PRE C: Premedication urine culture.

**Table 4:** The results of the urine test after treatment.

	Catheter Group (N = 84)		Non-Catheter Group (N = 344)	
	Success	Failure	Success	Failure
Turbidity	55 (65.5%)	29 (34.5%)	279 (81.1%)	65 (18.9%)
pH	82 (97.6%)	2 (2.4%)	344 (100.0%)	0 (0.0%)
WBC	37 (44.1%)	47 (56.0%)	200 (58.1%)	144 (41.9%)
RBC	29 (34.5%)	55 (65.5%)	185 (53.8%)	159 (46.2%)
Bacteria	45 (53.6%)	39 (46.4%)	242 (70.4%)	102 (29.7%)

**Table 5:** Treatment success rate according to characteristics.

	Catheter Group (N = 84)		Non-Catheter Group (N = 344)	
	Success	Failure	Success	Failure
Gender				
Male	16 (76.2)	5 (23.8)	51 (81.0)	12 (19.1)
Female	41 (65.1)	22 (34.9)	245 (87.2)	36 (12.8)
Age group				
19~29	2 (50.0)	2 (50.0)	19 (100.0)	0 (0.0)
30~19	4 (44.4)	5 (55.6)	41 (89.1)	5 (10.9)
50~64	13 (72.2)	5 (27.8)	59 (83.1)	12 (16.9)
65 or more	38 (71.7)	15 (28.3)	177 (85.1)	31 (14.9)
HTN				
HTN (−)	40 (66.7)	20 (33.3)	228 (89.1)	28 (10.9)
HTN (+)	17 (70.8)	7 (29.2)	68 (77.3)	20 (22.7)
DM				
DM (−)	19 (57.6)	14 (42.4)	159 (88.3)	21 (11.7)
DM (+)	38 (74.5)	13 (25.5)	137 (83.5)	27 (16.5)

**Table 6:** Antibiotic sensitivity according to catheter group.

		Catheter Group	Non-Catheter Group	*p*-Value
Vancomycin	R	2 (100%)	0 (0%)	0.254
	S	10 (62.5%)	6 (37.5%)	
Tobramycin	R	23 (74.2%)	8 (25.8%)	0.872
	S	67 (77.9%)	19 (22.1%)	
Tigecycline	R	0 (0%)	0 (0%)	0.006
	S	85 (82.5%)	18 (17.5%)	
Tetracycline	R	32 (76.2%)	10 (23.8%)	0.662
	S	34 (72.3%)	13 (27.7%)	
Trimethoprim	R	43 (82.7%)	9 (17.3%)	0.218
Sulfamethoxazole	S	48 (76.2%)	15 (23.8%)	
Piperacillin	R	56 (77.8%)	16 (22.2%)	0.403
	S	36 (80.0%)	9 (20.0%)	
Tazobactam	R	5 (83.3%)	1 (16.7%)	0.210
	S	87 (79.1%)	23 (20.9%)	
Minocycline	R	10 (76.9%)	3 (23.1%)	0.913
	S	43 (78.2%)	12 (21.8%)	
Meropenem	R	3 (100%)	0 (0%)	0.495
	S	92 (77.3%)	27 (22.7%)	
Levofloxacin	R	53 (76.8%)	16 (23.2%)	1.000
	S	52 (75.4%)	17 (24.6%)	
Imipenem	R	4 (100%)	0 (0%)	0.252
	S	89 (78.1%)	25 (21.9%)	
Gentamycin	R	44 (77.2%)	13 (22.8%)	1.000
	S	63 (75.9%)	20 (24.1%)	
Fosfomycin	R	4 (80.0%)	1 (20.0%)	0.816
	S	53 (79.1%)	14 (20.9%)	
Ertapenem	R	0 (0%)	0 (0%)	0.747
	S	52 (77.6%)	15 (22.4%)	
Doripenem	R	1 (100%)	0 (0%)	0.390
	S	92 (78.6%)	25 (21.4%)	
Cefuroxime	R	31 (70.5%)	13 (29.5%)	0.036
	S	53 (86.9%)	8 (13.1%)	
Cefepime	R	32 (72.7%)	12 (27.3%)	0.204
	S	63 (81.8%)	14 (18.2%)	
Ciprofloxacin	R	56 (76.7%)	17 (23.3%)	0.993
	S	48 (76.2%)	15 (23.8%)	
Colistin	R	1 (50.0%)	1 (50.0%)	0.16
	S	92 (81.4%)	21 (18.6%)	
Cefoxitin	R	9 (69.2%)	4 (30.8%)	0.049
	S	74 (83.1%)	15 (16.9%)	
Cefotaxime	R	33 (73.3%)	12 (26.7%)	0.015
	S	57 (86.4%)	9 (13.6%)	
Ceftazidime	R	33 (73.3%)	12 (26.7%)	0.145
	S	61 (82.4%)	13 (17.6%)	
Chloramphenicol	R	7 (53.8%)	6 (46.2 %)	0.083
	S	44 (83.0%)	9 (17.0%)	
Aztreonam	R	30 (69.8%)	13 (30.2%)	0.119
	S	61 (83.6%)	12 (16.4%)	
Amoxicillin	R	10 (76.9%)	3 (23.1%)	0.487
Clavulanic acid	S	41 (82.0%)	9 (18.0%)	
Ampicillin	R	76 (79.2%)	20 (20.8%)	0.254
	S	23 (76.7%)	7 (23.3%)	
Amikacin	R	3 (75.0%)	1 (25.0%)	0.553
	S	92 (78.0%)	26 (22.0%)	
Sulbactam	R	34 (77.3%)	10 (22.7%)	0.284
	S	36 (83.7%)	7 (16.3%)	

## Data Availability

The data that support the findings of this study are available on request from the corresponding author.
